# Safety Profiles of Ionic and Non-Ionic Contrast Agents Used in Percutaneous Coronary Intervention: Analysis of FAERS Data: Short title: Safety Profiles of Ionic and Non-Ionic Contrast Agents Used in Percutaneous Coronary Intervention

**DOI:** 10.31661/gmj.v15i.4162

**Published:** 2026-07-13

**Authors:** Shirin Alord, Farid Tagavi, Haleh Bodagh, Mehdi Maleki, Negar Jafari, Seyed Mohammad Hassan Adel

**Affiliations:** ^1^ Cardiovascular Research Center, Health Policy and Promotion Institute, Kermanshah University of Medical Sciences, Kermanshah, Iran; ^2^ Cardiovascular Research Center, Tabriz University of Medical Sciences, Tabriz, Iran; ^3^ Department of Cardiology, School of Medicine, Urmia University of Medical Sciences, Urmia, Iran; ^4^ Atherosclerosis Research Center, Ahvaz Jundishapur University of Medical Sciences, Ahvaz, Iran

**Keywords:** Contrast Agents, Percutaneous Coronary Intervention, FAERS, Adverse Events, Ionic Agents, Non-ionic Agents, Safety Profile

## Abstract

**Background:**

Contrast agents are used in percutaneous coronary intervention (PCI), yet differences in adverse event profiles between ionic and non-ionic agents remain incompletely characterized. This study aimed to evaluate patient demographics, adverse event patterns, and seriousness of outcomes associated with contrast agents reported in the U.S. FDA Adverse Event Reporting System (FAERS).

**Materials and Methods:**

Data were extracted from FAERS for reports involving contrast agents used during PCI, including both ionic (low- and high-osmolar) and non-ionic agents. Datasets were cleaned for duplicates and incomplete records, and product names, patient demographics, and adverse reactions were standardized. Descriptive statistics were calculated for continuous (age, weight) and categorical (sex, seriousness, adverse reactions) variables. Comparative analyses between ionic and non-ionic groups were performed using t-tests for continuous variables and chi-square tests for categorical outcomes. Data visualization was performed using Python libraries.

**Results:**

A total of 72 cases were identified, including 18 ionic (25%) and 54 non-ionic (75%) reports. Mean patient ages ranged from 52.8 to 71.0 years across individual agents, with weight varying from 69.7 to 92.9 kg. Adverse events differed by agent type: ionic agents were primarily associated with renal failure and hypersensitivity reactions, while non-ionic agents exhibited broader nephrotoxic and hemodynamic complications, including nephropathy toxic, anaphylaxis, and blood pressure decreases. All products were linked to serious outcomes, with non-ionic agents showing a significantly higher association with serious events (χ²(1, N=72) = 5.92, P = .015). No significant differences in age or weight were observed between ionic and non-ionic groups. Temporal trends varied by agent, with sporadic case reporting over the years; while Ultraist has shown increased trend till 2025.

**Conclusion:**

Ionic and non-ionic contrast agents used in PCI demonstrate distinct adverse event profiles. These findings show the importance of tailored patient monitoring and risk assessment when selecting contrast agents for PCI procedures.

## Introduction

Percutaneous coronary intervention (PCI) is a cornerstone procedure in the management of coronary artery disease, offering effective revascularization for patients with myocardial ischemia. Despite its widespread use and technological advancements, PCI is not devoid of potential complications. Among the most significant risks are contrast-induced nephropathy (CIN) and acute kidney injury (AKI), particularly concerning patients with pre-existing renal impairment. CIN contributes substantially to post-procedural morbidity and mortality, extending hospital stays and increasing healthcare costs (Al Saif et al., 2024).

In coronary interventions, contrast media (CM) are essential for visualizing coronary arteries, broadly categorized by ionic (e.g., ioxaglate) and non-ionic types (e.g., iopromide, iodixanol), which differ in osmolarity and chemical structure [1, 2, 3, 5]. Ionic CM are typically low-osmolar dimers like ioxaglate, while non-ionic CM include low-osmolar monomers/iohexol and iso-osmolar dimers/iodixanol, generally considered to have superior safety profiles regarding allergic reactions and potential thrombotic effects [2, 3, 5, 6, 7], though some studies suggest specific ionic agents might reduce stent thrombosis [7]. However, research indicates that both ionic and non-ionic CM can influence hemostasis, reducing thrombin potential but potentially triggering thrombus formation visible via angioscopy, particularly with certain non-ionic agents [3, 5, 6], and may contribute to contrast-induced nephropathy (CIN), especially in chronic kidney disease (CKD) patients, where iso-osmolar CM did not show a significant advantage over low-osmolar CM [4]. Furthermore, studies comparing prediction formulas for CIN post-PCI highlight the potential inaccuracy of the Cockcroft-Gault formula [8], focusing on disrupting calcium deposits rather than relying solely on CM choice. The choice of CM remains complex, balancing risks like allergic reactions, thrombus formation, CIN, and stent complications against potential benefits depending on the specific procedure, patient population, and agent characteristics.

Non-ionic contrast agents generally demonstrate a lower incidence of immediate adverse effects such as allergic reactions compared to ionic agents, although combining different types during a single procedure did not increase overall adverse events beyond using an ionic agent alone [2]. However, specific ionic agents like ioxaglate were strongly linked to higher rates of acute and subacute stent thrombosis [8], suggesting a complex relationship where ionic CM might paradoxically offer better protection against thrombosis in this context. Regarding thrombotic potential, both ionic and non-ionic CM significantly reduced thrombin potential during diagnostic angiography but showed variable effects on thrombus formation during PCI, with non-ionic iohexol potentially increasing visible thrombus post-procedure compared to ioxaglate [3, 5, 6]. For CIN prevention in CKD patients undergoing coronary angiography, while iso-osmolar CM showed a non-significant trend towards lower CIN risk than low-osmolar CM, the difference was often minimal and not clinically decisive, particularly when comparing non-ionic low-osmolar agents directly [4]. The predictive accuracy of GFR formulas for CIN risk also varies, with CKD-EPI potentially identifying at-risk patients better than Cockcroft-Gault in certain scenarios [8]. The optimal choice between ionic and non-ionic CM, and the role of different osmolarities, remains an area of ongoing investigation due to conflicting evidence and complex interactions with procedural steps like stenting and hemostasis. Despite widespread use of contrast agents in PCI, the comparative safety profiles of ionic and non-ionic agents remain incompletely understood, particularly regarding adverse events and serious outcomes. Existing evidence is conflicting, with some studies suggesting differences in nephrotoxicity, thrombotic risk, and allergic reactions, but large-scale, real-world data are limited. The FAERS database provides a unique opportunity to systematically evaluate reported adverse events across multiple agents and patient populations. Understanding these patterns can inform risk stratification, guide contrast agent selection, and ultimately improve patient safety during PCI procedures.

## Materials and Methods

### Data Source

Data for this study were obtained from the U.S. Food and Drug Administration Adverse Event Reporting System (FAERS), a publicly available database that collects spontaneous reports of adverse events associated with pharmaceutical products. We focused on cases related to contrast agents used during percutaneous coronary intervention (PCI), including both ionic and non-ionic agents. Data were extracted for all available fields, including patient demographics, suspect product information, reported adverse reactions, seriousness of outcomes, and report source. We only included cases where Ultravist, Omnipaque, Optiray, Isovue, and Hexabrix were used for percutaneous coronary intervention angiography.

### Data Preprocessing

The raw FAERS dataset was first cleaned to remove duplicate records and incomplete entries. The Suspect Product Names were standardized to ensure consistent naming across reports. Patient age was converted to numeric format, and weight was standardized to kilograms, converting from pounds where necessary. Categorical variables such as sex, seriousness of outcome, and report source were cleaned and harmonized. Adverse reactions listed as semicolon-separated strings were split into lists to allow for frequency analyses. Records were grouped by Suspect Product Names to enable comparative analyses across different contrast agents.

### Statistical Analysis

Descriptive statistics were calculated for each product, including mean, standard deviation, median, minimum, and maximum for continuous variables (age and weight), and frequency counts for categorical variables (sex, seriousness, and adverse reactions). The top 5 most frequent adverse reactions were identified for each agent. Comparative analyses between product groups were performed using appropriate statistical tests: t-tests or ANOVA for continuous variables and chi-square tests for categorical outcomes. All analyses were conducted in Python using the pandas, numpy, matplotlib, seaborn, and collections libraries. Visualizations were created to display distributions of age, sex, serious outcomes, and top reactions by product, facilitating a comprehensive overview of safety profiles.

## Results

**Figure-1:**
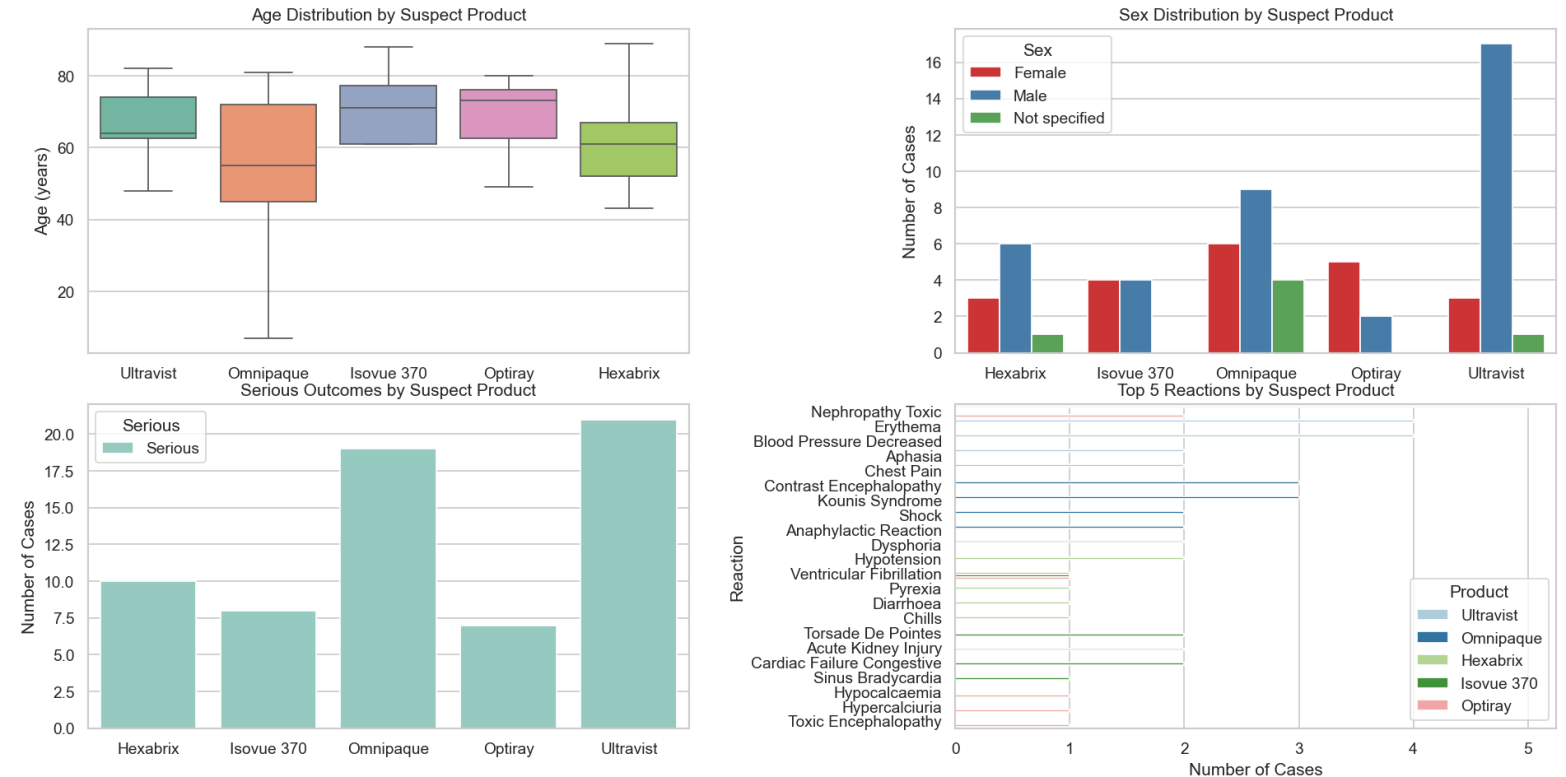
Figure 1. Descriptive analysis of adverse events associated with the five most frequently reported contrast agents used in PCI

**Figure-2:**
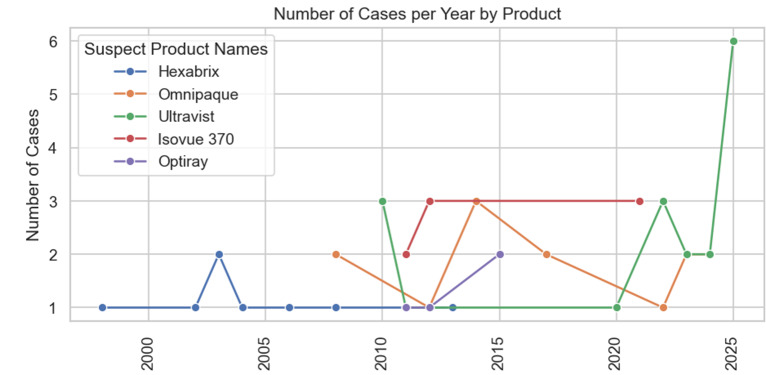
Figure 2. The annual distribution of adverse event cases reported to FAERS for the top five contrast agents used in percutaneous coronary interventions

**Table:** Table1. Patient Demographics by Contrast Type

Group	N (Age)	Age Mean (SD)	Median	Range	N (Weight)	Weight Mean (SD)	Median	Range
Ionic Low	12	66.42 (17.07)	65	43–93	4	69.73 (17.01)	69.46	55–85
Ionic High	6	56.83 (5.78)	56	51–67	3	73.07 (13.86)	73.5	59–86.7
Ionic All	18	63.22 (14.83)	60	43–93	7	71.16 (14.56)	73.5	55–86.7
Non-Ionic	54	65.09 (16.08)	65.5	7–88	31	77.47 (19.21)	75	52.61–139

**Table:** Table2. Sex Distribution and Serious Outcomes by Contrast Type

Group	Male	Female	Not Specified	Serious	Non-Serious
Ionic Low Smolar	7	5	2	14	0
Ionic High Smolar	3	3	0	3	3
Ionic All	10	8	2	17	3
Non-Ionic	36	21	5	62	0

**Table:** Table3. Case Outcomes, Priority, and Reporting Source

Group	Hospitalized	Died	Life-Threatening	Other Outcomes	Expedited	Direct	Non-Expedited	Healthcare Professional Reporter	Consumer/Other
Ionic Low	5	4	4*	1	11	2	1	8	6
Ionic High	0	0	2	3	0	1	5	4	2
Ionic All	5	4	5	2	11	3	6	12	8
Non-Ionic	17	8	11	25	55	1	6	49	11

^*^ Includes combinations of life-threatening with hospitalization or death.

### Sample Characteristics

A total of 72 cases of contrast agents used in percutaneous coronary intervention (PCI) were identified in the FAERS database. Among these, 18 cases (25%) involved ionic agents, subdivided into ionic low-osmolar (n = 12) and ionic high-osmolar (n = 6), while 54 cases (75%) involved non-ionic agents. Descriptive analysis of adverse events associated with the five most frequently reported contrast agents used in PCI is presented in Figure-1. Across the cohort, Ultravist (n=19) and Omnipaque (n=13) demonstrated mean patient ages of 67.1 ± 9.6 years and 52.8 ± 23.7 years, respectively, whereas Hexabrix (n=9), Isovue 370 (n=8), and Optiray (n=7) had mean ages ranging from 60.7 to 71.0 years. Weight distributions varied, with Omnipaque and Isovue 370 showing higher mean weights (92.9 ± 31.4 kg and 92.6 ± 14.0 kg) compared with Ultravist, Hexabrix, and Optiray. The majority of cases for all products occurred in males, except for Isovue 370 and Optiray, which had a roughly equal or female-predominant distribution. All products were associated with serious outcomes in all reported cases. The most commonly reported reactions differed by agent: Ultravist and Optiray were primarily linked to nephrotoxic events (e.g., nephropathy toxic), Omnipaque to neurologic or hypersensitivity reactions (contrast encephalopathy, Kounis syndrome), Hexabrix to hemodynamic disturbances (hypotension, ventricular fibrillation), and Isovue 370 to cardiac or renal complications (torsade de pointes, acute kidney injury). Figure-2 illustrates the annual distribution of adverse event cases reported to FAERS for the top five contrast agents used in percutaneous coronary interventions. Ultravist showed a total of 19 cases, with the highest number reported in 2025 (n=6), followed by smaller counts scattered across 2010, 2011, 2012, 2020, 2022, and 2024. Omnipaque accounted for 10 cases, with peaks in 2008 (n=3) and 2014 (n=4), while Hexabrix had 9 cases distributed from 1998 through 2013, showing small numbers per year without a clear temporal trend. Isovue 370 contributed 8 cases, primarily in 2011, 2012, and 2021, and Optiray had 5 cases occurring between 2011 and 2015.

Table1 summarizes patient demographics for age and weight. Analysis of the top reported adverse reactions revealed distinct patterns between ionic and non-ionic contrast agents. Among ionic low-osmolar agents, the most frequently reported reactions were renal failure (n = 3), diarrhoea (n = 2), and hypotension (n = 2), with less common reactions including ventricular fibrillation, pyrexia, chills, dehydration, vomiting, acute coronary syndrome, and renal tubular necrosis (each n = 1). For ionic high-osmolar agents, the most prevalent reactions were urticaria (n = 3) and anaphylactic shock (n = 2), followed by back pain, chills, and respiratory arrest (each n = 1). When combining all ionic agents, the pattern reflected a mixture of renal and hypersensitivity events: renal failure (n = 3), anaphylactic shock (n = 3), urticaria (n = 3), diarrhoea (n = 2), chills (n = 2), and hypotension (n = 2), with less frequent reactions including ventricular fibrillation, pyrexia, dehydration, and vomiting (each n = 1). In contrast, non-ionic agents demonstrated a markedly different profile, with nephropathy toxic (n = 10) as the most commonly reported reaction, followed by anaphylactic reaction (n = 5) and blood pressure decreased (n = 5). Other notable reactions included erythema and contrast encephalopathy (n = 4 each), acute kidney injury (n = 4), Kounis syndrome and cardiac arrest (n = 3 each), and aphasia and tachycardia (n = 2 each). Overall, this comparison highlights that ionic agents are more frequently associated with renal failure and hypersensitivity reactions, whereas non-ionic agents exhibit broader nephrotoxic and hemodynamic adverse events, underscoring distinct safety profiles between these contrast agent classes. No significant differences in age or weight were observed between Non-Ionic (M = 65.09, SD = 16.08; M = 77.47, SD = 19.21) and Ionic All groups (M = 63.22, SD = 14.83; M = 71.16, SD = 14.56) using independent-samples t-tests: age, t(69) = 0.45, P = .653; weight, t(36) = 0.94, P = .351. Table2 presents the sex distribution and serious outcomes across contrast types. A chi-square test indicated a significant association between contrast type (Ionic vs Non-Ionic) and serious outcomes, χ²(1, N = 72) = 5.92, P = .015, suggesting that non-ionic agents were more frequently associated with serious adverse events. Table3 shows outcomes, case priority, reporter type, and report source for each group.

## Discussion

Our study utilized post-market FAERS data to compare the safety profiles of ionic and non-ionic contrast agents in a relatively small cohort (N=72) undergoing PCI. While our analysis identified distinct adverse event patterns, with non-ionic agents showing a significantly higher association with serious events (χ²(1, N=72) = 5.92, P = .015) compared to ionic agents, it lacked the power to explore potential risk factors or adjust for confounding variables inherent in real-world data. Furthermore, studies like TRUST study [9] provide valuable large-scale real-world data on the safety of specific agents like iopromide, reporting a very low incidence of acute adverse drug reactions in cardiac catheterization, though often focusing solely on immediate reactions (within 1 hour) rather than the broader range of potential adverse events captured in passive surveillance like FAERS. The TRUST study [4], being a large real-world registry, supports the feasibility and safety of common ionic agents but focuses on a different time frame and specific agent, limiting direct comparison. Our study’s inclusion of non-CIN related serious events and hypersensitivity/hemodynamic issues adds valuable depth beyond renal-focused analyses [9].

While contrasting our findings, the study by Juergens et al. [10] investigated allergic reactions and MACE in a clinical PCI setting using specific agents (ioxaglate and iopromide). They found no significant increase in MACE or allergic reactions (even with combined use) compared to iopromide alone in their patient population, although ioxaglate alone was strongly associated with allergies (P = 0.021). This suggests that in a controlled clinical setting, the specific agents used might yield different results than real-world spontaneous reporting captured by FAERS, particularly regarding allergy incidence. Clauss et al. [11] demonstrated in a porcine restenosis model that incubation with iopromide, ioxaglate, or iosimenol did not affect bovine aortic smooth muscle cell proliferation or subsequent in-stent neointimal hyperplasia or restenosis. Similarly, Danzi et al. [12], in a randomized clinical trial with over 1300 patients undergoing coronary stenting, found no significant differences among ioxaglate, iopamidol, and iopromide regarding 30-day major adverse cardiac events (MACCE), although ioxaglate showed a significantly higher rate of overall adverse reactions (P = 0.001). Collectively, their studies [11, 12] suggest that, under experimental and short-term clinical follow-up conditions, the choice between these modern contrast media might not significantly impact long-term procedural outcomes like restenosis or MACCE, except for noted allergic or other adverse reactions, areas where your FAERS study highlighted distinct patterns and seriousness between ICA and NICA types.

## Conflict of Interest

The authors declare no conflict of interest.

## AI Disclosure Statement

During the preparation of this manuscript, the authors used ChatGPT, OpenAI company for language editing, grammar improvement, and liboberry.com for reference management. After its use, the authors thoroughly reviewed, verified, and revised all AI-assisted content to ensure accuracy and originality. The authors take full responsibility for the integrity and final content of the published article.
